# Exploring the challenges of AI-driven business intelligence systems in the Malaysian insurance industry

**DOI:** 10.12688/f1000research.163354.1

**Published:** 2025-04-22

**Authors:** Sharmila Ramachandaran, Zubaidi Mahalley, Riska Nuraini, Bablu Kumar Dhar

**Affiliations:** 1 Faculty of Business and Communication, INTI International University & Colleges, Nilai, Negeri Sembilan, Malaysia; 2 INTI International University & Colleges, Nilai, Negeri Sembilan, Malaysia; 3 Business Admistration Division, Mahidol University International College, Mahidol University, Salaya, Nakhon Pathom, Thailand

**Keywords:** Artificial Intelligence, Business Intelligence, Malaysian Insurance Industry, Technology-Organization-Environment Framework, Resource-Based View, Digital Transformation, Emerging Markets

## Abstract

**Background:**

Integrating Artificial Intelligence (AI) with Business Intelligence (BI) systems in the insurance industry holds the potential for enhanced operational efficiency, strategic decision-making, and improved customer experiences. However, the Malaysian insurance sector faces numerous challenges in realizing this potential, including organizational resistance, skill shortages, regulatory complexities, and financial constraints. This study explores the specific challenges encountered in the adoption of AI-driven BI systems within the Malaysian insurance industry.

**Methods:**

Using an integrated framework that combines the Technology-Organization-Environment (TOE) model and Resource-Based View (RBV), this research examines the internal and external factors that impact AI adoption. A qualitative case study approach was employed, involving in-depth interviews with technical experts, middle management, and senior leaders from key industry players. Thematic analysis of the data identified significant barriers to AI adoption, such as organizational resistance, lack of skilled personnel, and the complexities of navigating regulatory frameworks.

**Results:**

The findings provide a deep understanding of the key challenges faced by Malaysian insurers and highlight areas that require attention, such as leadership commitment, workforce upskilling, technological infrastructure improvements, and policy advocacy.

**Conclusion:**

This study adds to the limited academic literature on AI-driven BI adoption in emerging markets and offers practical insights to insurers for overcoming these challenges. By addressing these obstacles, this research contributes to the broader discourse on digital transformation in the insurance sector, offering valuable recommendations for overcoming hurdles in AI adoption while maintaining compliance and ensuring customer-centric approaches.

## Introduction

Artificial intelligence (AI) is rapidly transforming industries worldwide, and its integration with business intelligence (BI) is revolutionising how organisations leverage data for strategic decision-making. AI-powered business intelligence solutions systems, employing advanced algorithms, machine learning, and predictive analytics, offer unprecedented capabilities to analyse vast datasets, uncover hidden patterns, and generate actionable insights with speed and accuracy surpassing traditional BI approaches. This transformative potential is particularly relevant in the insurance industry, where vast amounts of data, from customer demographics and claims history to market trends and risk assessments, are key to enhanced operational efficiency, improved customer experiences, and more effective risk management.

However, adopting AI-powered business intelligence solutions within the Malaysian insurance sector must meet these global trends. While the Malaysian government has launched initiatives to promote AI adoption across various sectors, the insurance industry faces unique challenges, including regulatory complexities, a shortage of skilled talent, and cultural perceptions surrounding AI. This study aims to explore the current state of AI-powered business intelligence solutions within the Malaysian insurance industry and identify the key challenges hindering their wider adoption.

### Problem statement

Despite the global advancements and proven benefits of AI-powered business intelligence solutions in the insurance industry, the Malaysian insurance sector still needs to fully embrace these technologies. The slow adoption rate poses significant risks, including decreased competitiveness, inability to meet evolving customer expectations, and operational inefficiencies that could impact profitability and sustainability ( Boston Consulting Group (BCG), 2023).

The global digital transformation underscores the urgency of the problem. Customers increasingly demand personalised, seamless digital experiences, and insurers that fail to deliver may lose market share to more agile competitors, including InsurTech startups and foreign insurers operating in Malaysia ( Ernst and Young, 2017). Moreover, operational inefficiencies resulting from reliance on legacy systems hinder the ability of Malaysian insurers to optimise costs and respond swiftly to market changes.

Evidence of the problem is highlighted in a study conducted by Boston Consulting Group ( BCG, 2023). Malaysian companies trail their global and regional peers in digital transformation, with only 17% adequately set up for digital transformation success. Embedded AI adoption, in particular, is significantly lagging, with Malaysian companies scoring an average of 4.7 on AI adoption compared to Southeast Asia’s 7.1 and global averages of 7.4. A report by the Asian Institute of Insurance ( SAS Institute Inc., 2024) found that only 15% of insurers have integrated AI-powered business intelligence solutions into their core operations. Key challenges identified include organisational resistance to change, lack of skilled talent, inadequate technological infrastructure, and regulatory complexities.

The slow adoption of AI-powered business intelligence solutions impacts stakeholders such as customers, employees, shareholders, and regulators. Customers may experience suboptimal service levels, employees may face increasing workloads due to inefficient processes, and shareholders may see diminished returns. While aiming to ensure stability and consumer protection, regulators recognize the need to balance oversight with facilitating innovation ( The Edge Malaysia, 2022).

Addressing these challenges aligns with Malaysia's national objectives of becoming a high-income nation and a leader in the digital economy, as outlined in the MyDIGITAL blueprint ( Economic Planning Unit, 2021). Therefore, it is critical to investigate the factors impeding the adoption of AI-powered business intelligence solutions, assess the effectiveness of current implementations, and develop strategies tailored to the Malaysian context.

### Research objectives

• To identify the challenges affecting the adoption of AI-driven business intelligence in the Malaysian insurance sector.

### Significance of the research

This research holds significant implications across practical, academic, and policy spheres. From a practical standpoint, the findings of this study will provide valuable insights for insurance companies operating in Malaysia. By identifying the specific challenges hindering the adoption of AI-powered business intelligence solutions, this research will empower insurers to develop targeted interventions and overcome these obstacles.

Academically, this study contributes to the growing knowledge surrounding AI adoption in emerging markets. It addresses a significant gap in the literature by focusing specifically on the Malaysian insurance sector, providing empirical data and insights that can inform future research and theoretical development. The study also extends the application of existing theoretical frameworks, such as the Technology-Organization-Environment (TOE) model, to a new context, further enriching our understanding of the factors influencing technology adoption.

Finally, this research carries important policy implications. By highlighting the challenges and opportunities associated with AI-powered business intelligence solutions, the study will inform policymakers and regulators in Malaysia about the necessary steps to create a supportive regulatory environment. This includes developing guidelines and frameworks for ethical AI use in insurance and ensuring consumer protection while fostering innovation. The research will also offer recommendations for aligning national policies with industry needs, accelerating the digital transformation of the Malaysian insurance sector and contributing to the nation's broader economic goals.

## Literature review

### Introduction

The advent of Artificial Intelligence (AI) is rapidly transforming the insurance sector, presenting both opportunities and challenges for companies seeking to leverage its power. Integrating AI-driven Business Intelligence (BI) systems in Malaysia is particularly compelling, promising enhanced operational efficiency, improved customer experiences, and a competitive edge in a dynamic market. However, realising this potential requires navigating a complex technological, organisational, and regulatory landscape. This chapter provides a comprehensive literature review exploring the theoretical underpinnings, challenges, and potential strategies for adopting successful AI-powered business intelligence solutions within the Malaysian insurance industry, emphasising the challenges faced.

### Theoretical frameworks and models

This chapter examines theoretical frameworks for adopting AI-driven Business Intelligence (BI) in the Malaysian insurance industry. We analyse key theories, assess their applicability to this context, and highlight their strengths and weaknesses. This analysis justifies the adoption of an integrated framework combining the Technology-Organization-Environment (TOE) and Resource-Based View (RBV) for this study.

**Technology-Organization-Environment (TOE) framework**

The TOE framework ( Tornatzky, Fleischer and Chakrabarti, 1990) provides a more comprehensive organisational-level analysis, considering Technological, Organizational, and Environmental contexts. This framework is particularly suitable for analysing the adoption of AI-powered business intelligence solutions in Malaysian insurance, as it encompasses the multifaceted factors influencing organisational decisions. However, it doesn't explicitly address the development and leveraging of internal resources for competitive advantage.

**Resource-Based View (RBV)**

The RBV ( Barney, 1991) emphasises internal resources and capabilities as sources of competitive advantage. Applied to AI-powered business intelligence solutions, RBV highlights the importance of developing unique AI capabilities and fostering an innovative culture. However, RBV alone may not fully capture the influence of external pressures and technological change, which are significant in the rapidly evolving field of AI.

**Integrated TOE-RBV framework**

This study adopts an integrated TOE-RBV framework to address the limitations of individual models. This framework provides a holistic approach, considering both external influences (TOE) and internal capabilities (RBV). This is crucial for understanding the adoption of AI-powered business intelligence solutions in Malaysian insurance, where factors like technological infrastructure, organisational culture, regulatory compliance, and strategic alignment are interconnected.

*Challenges of AI-Driven BI Adoption in the Malaysian Insurance Sector*

Adopting AI-powered business intelligence solutions in the Malaysian insurance industry presents several challenges that can hinder effective implementation. This section thoroughly explores these challenges, drawing on empirical studies, industry reports, and theoretical perspectives.

## Organizational resistance

### Cultural factors

Organisational culture plays a significant role in technology adoption. In Malaysia, many organisations exhibit hierarchical structures with centralised decision-making processes ( Abd Rahman, Kamarulzaman and Sambasivan, 2013). Such structures can lead to resistance to change, as decisions are concentrated at higher levels, and employees may feel disengaged from innovation initiatives.

As described by Hofstede's cultural dimensions, the national culture is characterised by high power distance ( The Cultural Factor, 2023). High power distance indicates acceptance of unequal power distribution, which may suppress open communication and feedback.

The survey conducted by Oppotus in 2023 suggested that only 25% of respondents are actively using AI applications and highlights the scepticism regarding AI’s benefits and implications for job security ( Oppotus, 2023). Employees may fear AI technologies disrupting established workflows, threatening job security, or requiring significant role adjustments. This fear can manifest as passive resistance, reluctance to engage with new systems or active opposition to adoption efforts.

### Change management issues

Effective change management is crucial for successful technology adoption. Lack of clear communication, inadequate training, and insufficient involvement of employees in the adoption process contribute to resistance ( Saghafian, Laumann and Skogstad, 2021).

Leadership commitment is essential in guiding organisations through change. Nguyen and Waring (2013) emphasised that transformational leadership, which inspires and motivates employees, can reduce resistance by fostering a shared vision and encouraging participation.

In the Malaysian insurance sector, resistance may intensify if leaders do not actively support AI-powered business intelligence solutions initiatives or fail to address employee concerns. Employees may perceive the change as imposed rather than collaborative, leading to decreased morale and adoption rates ( Abd Rahman, Kamarulzaman and Sambasivan, 2013).

## Skill gaps and talent shortages

### Current talent landscape

The successful implementation of AI-powered business intelligence solutions hinges critically on the availability of a skilled workforce proficient in AI, machine learning, data science, and analytics. However, Malaysia currently needs more professionals with these specialised skills. The Malaysia Digital Economy Corporation (MDEC) (2024) estimates that the demand for data scientists and AI specialists outstrips the current supply by a staggering 60%. This talent deficit poses a significant challenge for Malaysian insurance companies seeking to adopt and effectively utilise AI-powered business intelligence solutions.

This shortage stems from a confluence of factors. One key contributor is the gap between the skills produced by educational institutions and the practical needs of the industry ( Bujang et al., 2020). While Malaysian universities offer computer science and information technology programs, the curricula often need a more specialised focus on AI and data analytics, which are required in today's rapidly evolving technological landscape. This results in a mismatch between theoretical knowledge and the practical skills needed to develop, implement, and manage AI-powered business intelligence solutions systems.

Another contributing factor is the phenomenon of brain drain ( Abidin et al., 2022). Skilled professionals in Malaysia, particularly those with expertise in high-demand areas like AI, are often lured by more attractive opportunities abroad, where salaries and career prospects may be significantly better. This talent outflow exacerbates the skills shortage within the Malaysian insurance sector.

Finally, the rapid pace of technological advancements in AI presents a continuous challenge ( Moats, 2021). The field is evolving so quickly that it is difficult for educational institutions and training programs to keep up. This makes it challenging to equip professionals with the latest skills and knowledge needed to work with cutting-edge AI technologies, further contributing to the talent gap in the Malaysian insurance industry.

### Education and training

Addressing the skills gap in AI and data analytics requires a concerted effort to enhance education and training opportunities within Malaysia. A crucial step is fostering closer collaboration between educational institutions and the insurance industry to develop curricula aligned with practical industry needs. This means equipping graduates with theoretical knowledge and the hands-on skills to create, implement, and manage AI-powered business intelligence solutions systems. However, even with such collaborations, several challenges persist.

Resource constraints pose a significant obstacle for many Malaysian universities ( Aithal and Maiya, 2023). Developing and delivering high-quality AI programs requires substantial investment in qualified faculty, advanced technology, and robust industry partnerships. Many institutions may need more resources to offer cutting-edge programs that can effectively prepare students for the demands of the AI-driven workplace.

Another challenge lies in the limited availability of professional training opportunities, particularly for those already working in the insurance sector ( Petridou and Lao, 2024). Continuing education and professional development programs in AI and data analytics are essential for upskilling the existing workforce and enabling them to adapt to the changing technological landscape. However, access to such programs still needs to be improved, hindering the ability of insurance professionals to acquire the necessary skills to utilise AI-powered business intelligence solutions effectively. This underscores the need for greater investment in accessible and relevant training programs that cater to the specific needs of working professionals in the Malaysian insurance industry.

### Government initiatives

The Malaysian government has recognised the importance of building digital talent. It has launched initiatives like the #mydigitalmaker Movement and partnerships with technology companies to promote digital skills among students and professionals ( MDEC, 2024).

Additionally, the National Policy on Industry 4.0 (Industry4WRD) aims to transform Malaysia's manufacturing sector through advanced technologies, including AI and emphasises human capital development ( Ministry of Investment, Trade and Industry (MITI) (2022).

Despite these efforts, the impact on the insurance sector may be limited due to the specific skill requirements and the need for industry-specific training.

## Technological infrastructure

### Legacy systems

A significant impediment to adopting AI-powered business intelligence solutions in the Malaysian insurance sector is the prevalence of legacy IT systems. As noted by Tambi and Dahlan (2023), many insurance companies in Malaysia still rely on systems that have been in place for decades. These outdated systems often need interoperability and are compatible with modern AI technologies, creating a major roadblock to innovation.

The challenges associated with legacy systems are multifaceted. Integrating AI-powered business intelligence solutions with these older systems requires substantial technical effort, often involving extensive customisation and potentially even a complete re-engineering of existing processes ( Broby, 2021; Mahmood, Khan and Bokhari, 2020). This complexity translates into significant costs and time investments, deterring many insurers from pursuing AI-powered business intelligence solutions initiatives.

Furthermore, legacy systems often contribute to the problem of isolated systems for data storage (commonly referred to as 'data silos') ( Kothandapani, 2024). Information is stored in isolated systems, making gaining a holistic view of the data difficult, if not impossible. This fragmentation hinders the comprehensive data analysis essential for effective AI-powered business intelligence solutions. AI algorithms thrive on large, integrated datasets, and the presence of isolated systems for data storage (commonly referred to as 'data silos') severely limits their ability to uncover meaningful insights.

Finally, legacy systems' ongoing maintenance and upgrading represent a substantial drain on resources ( Broby, 2021). These costs divert valuable funds away from investments in innovation and modernisation, further hindering the adoption of AI-powered business intelligence solutions. The financial burden of maintaining outdated systems creates a vicious cycle, trapping insurers in a technological status quo and preventing them from realising the full potential of AI.

### Data quality and integration

High-quality, integrated data is the lifeblood of effective AI-powered business intelligence solutions. However, Malaysian insurers frequently need help with data quality and integration challenges, hindering their ability to leverage AI effectively. These challenges manifest in several ways.

One common issue is the inconsistency of data formats. Data collected over time and from various sources varies in format, structure, and standards. This lack of uniformity makes integrating data from different systems difficult and creates significant challenges for AI algorithms, which require consistent and standardised data for optimal performance.

Effective data governance also needs to be improved in many organisations. Data quality needs robust data governance policies and procedures, leading to inaccuracies, duplication, and potential security vulnerabilities. Data governance needs to be improved to ensure the reliability of AI-driven insights, which can lead to flawed decision-making.

Another significant hurdle is the limited availability of relevant data. Insufficient historical data or a lack of real-time data feeds can severely restrict the effectiveness of AI models. AI algorithms, particularly those based on machine learning, require large volumes of data to train effectively and generate accurate predictions. If the available data is limited or complete, the performance of AI-powered business intelligence solutions systems will be protected. As Ibrahim, Mohamed, and Satar (2021) emphasise, poor data quality has a detrimental impact on the success of data analytics initiatives, which holds for AI-powered business intelligence solutions as well. Addressing these data-related challenges is crucial for unlocking the full potential of AI in the Malaysian insurance sector.

### Technological readiness

Technological readiness, or the capacity of an organisation to adopt and effectively utilise new technologies, varies significantly among Malaysian insurers. As Ching, Teoh, and Amran (2020) observe, more prominent firms generally possess more advanced IT capabilities than their smaller counterparts. This disparity in technological readiness stems from several key factors.

Financial resources play a crucial role. Larger insurance companies typically have greater access to capital, allowing them to invest more heavily in technology upgrades and infrastructure development. This financial advantage enables them to acquire and implement advanced AI-powered business intelligence solutions systems more readily than smaller firms with limited budgets.

Strategic priorities also influence technological readiness. Firms prioritising innovation and digital transformation are more likely to allocate resources towards AI-powered business intelligence solutions and other emerging technologies. This proactive approach positions them to capitalise on the opportunities presented by AI and gain a competitive edge in the market.

Finally, the availability of in-house IT expertise is critical to technological readiness. Organisations with a skilled IT workforce are better equipped to implement and manage complex AI-powered business intelligence solutions systems. They possess the technical know-how to integrate these systems with existing infrastructure, troubleshoot technical issues, and ensure the smooth operation of AI-powered solutions. The lack of such expertise can be a major barrier to adoption for smaller insurers, highlighting the importance of investing in talent development and acquisition.

## Regulatory and compliance issues

### Data privacy laws

Data privacy regulations play a crucial role in shaping the adoption and implementation of AI-powered business intelligence solutions in Malaysia. The Personal Data Protection Act (PDPA) (2010) sets the legal framework for processing personal data in commercial transactions, imposing specific obligations on organisations that collect and utilise such data. While designed to protect consumer privacy, these regulations can present challenges for AI-powered business intelligence solutions initiatives, which often rely on analysing large datasets containing sensitive customer information.

The PDPA mandates that organisations obtain explicit consent from individuals before collecting and processing their personal data. This consent requirement necessitates transparent data collection practices and clear communication with customers about how their data will be used. Furthermore, the principle of purpose limitation stipulates that data can only be collected for specific, legitimate purposes and cannot be processed in any manner incompatible with those purposes. This restriction requires insurers to carefully define the scope of their data collection and usage in AI-powered business intelligence solutions initiatives.

The PDPA also places a strong emphasis on data security. Organisations are legally obligated to implement appropriate measures to safeguard personal data from loss, misuse, or unauthorised access. This includes implementing robust security protocols, encryption technologies, and access controls to protect the confidentiality and integrity of customer data. Compliance with these data security requirements can be complex and costly, particularly for smaller insurers with limited resources. Therefore, navigating the regulatory landscape of data privacy is crucial for insurers seeking to implement AI-powered business intelligence solutions systems in Malaysia. They must ensure that their data processing activities fully comply with the PDPA to avoid legal risks and maintain customer trust.

### Regulatory uncertainty

While Bank Negara Malaysia (BNM) (2022) encourages innovation in the financial sector, including adopting fintech and InsurTech solutions, the lack of specific guidelines on AI usage in insurance creates a climate of regulatory uncertainty. This ambiguity poses a significant challenge for insurers considering investments in AI-powered business intelligence solutions.

The absence of clear regulations leads to a cautious approach among many insurers. Concerned about potential non-compliance or unforeseen future regulatory changes, companies may hesitate to fully embrace AI-powered business intelligence solutions. This hesitancy can stifle innovation and prevent insurers from realising the potential benefits of AI.

Furthermore, this regulatory uncertainty increases the risk of legal challenges and penalties. Insurers need clear guidelines on data protection, algorithmic bias, and ethical considerations in AI deployment to avoid inadvertently violating regulations. This legal ambiguity can be a significant deterrent, particularly for smaller insurers with limited resources to navigate complex legal landscapes.

Globally, the regulatory landscape for AI/ML in finance is still nascent, with various jurisdictions adopting different approaches. While some economies like Singapore, Hong Kong, and the UK have published high-level principles or issued guidance on best practices ( BNM, 2022), Malaysia's regulatory framework still needs to be developed. More specific guidance on model interpretability and explainability further complicates the adoption of AI-powered business intelligence solutions in the Malaysian insurance sector. Insurers must, therefore, carefully consider these regulatory uncertainties and adopt a proactive approach to risk management when implementing AI solutions. This includes staying informed about global regulatory developments, engaging in dialogue with regulators, and implementing robust internal governance frameworks to mitigate potential risks.

### Compliance costs

Meeting regulatory requirements, particularly those related to data privacy and security, entails significant financial and operational costs for insurance companies. These compliance costs can pose a substantial barrier to adopting AI-powered business intelligence solutions, especially for smaller insurers with limited resources ( Hamid et al., 2022).

Investing in robust compliance infrastructure is a significant expense. Insurers must implement systems and controls to monitor and ensure compliance with data protection regulations. This may involve purchasing specialised software, upgrading existing IT infrastructure, and hiring dedicated compliance personnel. These investments can strain the budgets of smaller insurers, making it difficult to justify the additional expense of AI-powered business intelligence solutions.

Training personnel on legal obligations and compliance procedures is another significant cost. Employees need to be educated on the intricacies of data privacy laws, ethical AI practices, and internal compliance protocols. This requires developing and delivering training programs, which can be time-consuming and resource intensive.

Finally, engaging legal and consulting experts to navigate the complex regulatory landscape can significantly reduce compliance costs. Seeking external advice on data protection, regulatory compliance, and ethical AI practices is often necessary, but it comes at a price. These professional fees can be substantial, increasing the financial burden of adopting AI-powered business intelligence solutions. Therefore, insurers must carefully consider these compliance costs when evaluating the feasibility of AI-powered business intelligence solutions initiatives. They need to factor in the initial investment in technology and the ongoing expenses associated with meeting regulatory requirements.

## Method

### Research design

This study adopts a qualitative research methodology to explore the complexities and nuances of adopting AI-powered business intelligence solutions in the Malaysian insurance industry. Qualitative research is appropriate when the goal is to understand phenomena from the perspectives of those experiencing them, allowing for exploring meanings, experiences, and interpretations ( Creswell, 2013). The study's exploratory nature necessitates a methodology that can capture the depth and richness of participants' insights regarding the effectiveness of AI-powered business intelligence solutions systems.

Within the qualitative paradigm, a case study design is particularly suitable for this research. A case study, according to Yin (2018), is an empirical investigation that examines a current phenomenon in its actual context, particularly when the lines separating the phenomenon and setting. This aligns with the study's aim to examine AI-powered business intelligence solutions within the specific context of the Malaysian insurance industry. Stake (1995) emphasises that case studies allow for exploring the particularity and complexity of a single case, capturing the activity in essential circumstances.

### Participant selection and sampling strategy

The study aims to include a purposive sample of nine participants across different organisational levels within the Malaysian insurance industry. Specifically, the sample will comprise approximately two or three participants from the following groups: technical specialists, middle management, and senior management. This stratification allows for exploring diverse perspectives on the effectiveness of AI-powered business intelligence solutions.

Participants will be selected from three to four insurance companies operating in Malaysia, encompassing both life and general insurance providers. Including multiple companies enhances the transferability of the findings by capturing variations across different organisational contexts ( Miles, Huberman and Saldaña, 2014). Companies will be chosen based on criteria such as significant market share, a history of at least five years of operation, and active implementation of AI-powered business intelligence solutions systems.

Sampling criteria for participants include:

• Technical Specialists: At least two years of experience working directly with AI-powered business intelligence solutions systems, involvement in day-to-day technical operations, and expertise in data analytics or related fields.
• Middle Management: Roles that involve overseeing departments where AI-powered business intelligence solutions is integrated, responsibility for operational decision-making, and at least five years of industry experience.
• Senior Management: Positions such as CEOs, CIOs, or directors with strategic oversight of AI initiatives, involvement in organisational decision-making at the highest level, and substantial experience in the insurance sector.

The sample size is justified based on qualitative research standards, where depth of information is prioritised over breadth. Guest, Bunce, and Johnson (2006) suggest that data saturation often occurs within the first 12 interviews when participants are relatively homogeneous regarding their experiences with the phenomenon under study. Creswell (2013) recommends a sample size of 5 to 25 for case studies, while Mason (2010) notes that saturation in qualitative studies typically occurs within 15 interviews. Given the stratified nature of the sample and the focus on different organisational levels, the proposed sample size is sufficient to achieve saturation and provide comprehensive insights.

The study employed a manual approach to data analysis. The extended data includes an interview guide, which provides a structured framework outlining key themes and questions used during data collection. Additionally, a participant information sheet was provided to participants, detailing the study’s purpose, procedures, and confidentiality measures. A consent form, signed by participants before the interviews, ensured informed participation. Furthermore, selected excerpts from manually transcribed and coded interview data demonstrate the thematic analysis process used in the study. These materials were integral to the data analysis and have been included in the extended data repository.

### Data collection procedures

Data collection will proceed through a three-phase approach, aligning with the participant groups:

Phase 1: Technical Specialists

The first phase involves conducting in-depth interviews with technical specialists. This phase establishes a foundational understanding of how AI-powered business intelligence solutions systems function within daily operations. Technical specialists can provide detailed insights into the practical challenges and successes of implementing AI technologies, data management practices, and the technical effectiveness of BI tools.

Interviews will explore system usability, integration with existing technologies, data quality issues, and perceptions of how AI-powered business intelligence solutions affect operational tasks. This phase sets the stage for understanding the technical underpinnings influencing organisational experiences with AI-powered business intelligence solutions.

Phase 2: Middle Management

The second phase focuses on middle management, which bridges the gap between technical teams and senior leadership. Interviews with middle managers will explore how AI-powered business intelligence solutions are integrated into operational workflows, their impact on decision-making processes, and the challenges faced in aligning technical capabilities with business objectives.

This phase examines themes such as change management, employee adoption, performance metrics, and operational efficiencies. Middle managers can provide insights into the organisational dynamics that facilitate or hinder the effective use of AI-powered business intelligence solutions systems.

Phase 3: Senior Management

The final phase involves interviews with senior management to gain strategic perspectives on the impact of AI-powered business intelligence solutions. Senior leaders can discuss the rationale behind AI investments, expectations regarding competitive advantage, and the perceived return on investment.

Topics will include strategic alignment of AI initiatives with organisational goals, challenges in implementing AI at a strategic level, and visions for the future role of AI-powered business intelligence solutions in the insurance industry. This phase completes the comprehensive exploration by connecting technical and operational insights to overarching organisational strategies.

The sequential approach allows for integrating findings from each phase, building a holistic understanding of the effectiveness of AI-powered business intelligence solutions across organisational levels.

### Data analysis plan

Data analysis will follow a thematic analysis approach as Braun and Clarke (2006) described. The process involves several steps:

1. Familiarization with Data: Interviews will be transcribed verbatim to ensure accuracy. The researcher will read and re-read transcripts, noting initial observations and reflections.
2. Generating Initial Codes: Transcripts were coded line-by-line, identifying meaningful data units related to the research objectives. The researcher created a codebook, grouping similar segments.
3. Searching for Themes: Coded data were reviewed to identify themes, which are broader patterns that capture significant aspects of the phenomenon under study.
4. Reviewing and Refining Themes: Themes were refined, merged, or discarded based on their relevance to the research questions and the richness of supporting data.
5. Defining and Naming Themes: The final set of themes was clearly defined and named, providing a logical framework for the findings.
6. Integrating Findings: Themes were related to the research objectives, theoretical frameworks, and the literature, allowing for interpretation and contextualisation.

The researcher maintained reflexive notes throughout this manual coding process to track analytical decisions and ensure transparency ( Lincoln and Guba, 1985).

### Justification for manual analysis

Choosing a manual coding approach rather than software-assisted analysis allowed for a more hands-on, reflective engagement with the data. While software tools can be efficient, manual analysis can enhance the researcher’s intuitive grasp of the data and foster a more profound interpretative process ( Sandelowski, 1995).

Moreover, manual analysis ensured that the researcher’s contextual knowledge, cultural sensitivity, and theoretical insights informed the coding and theme development, ultimately contributing to a richer and more nuanced analysis.

### Issues of trustworthiness

Ensuring trustworthiness in qualitative research involves addressing credibility, transferability, dependability, and confirmability ( Lincoln and Guba, 1985).

• Credibility: Credibility refers to confidence in the truth of the findings. I will use triangulation to enhance credibility by collecting data from multiple sources (technical specialists, middle management, senior management) and comparing perspectives. Member checking will be conducted by sharing summaries of findings with participants to verify accuracy and resonance with their experiences.
• Transferability: Transferability pertains to how findings can be applied to other contexts. By providing rich, thick descriptions of the research context, participants, and findings, readers can assess the applicability of the results to different settings. Including multiple insurance companies and diverse participant roles enhances the potential for transferability.
• Dependability: Dependability involves demonstrating that the research process is logical, traceable, and documented. An audit trail will be maintained, detailing all aspects of the research process, including data collection procedures, coding decisions, and theme development. Peer debriefing will also contribute to dependability by subjecting the research process to external scrutiny.
• Confirmability: Confirmability ensures that the participants rather than researcher bias shapes the findings. Reflexivity practices, such as maintaining a reflexive journal and engaging in self-awareness, will help mitigate personal biases. The audit trail will also document decisions made throughout the research, providing transparency.

### Ethical procedures

Ethical considerations are critical, particularly given the involvement of human participants and the sensitivity of organisational information in the insurance industry.

• Informed Consent: Participants received an information sheet outlining the study's purpose, procedures, potential risks, and benefits. Written informed consent was obtained before participation, ensuring that participants were fully aware of their rights, including the right to withdraw at any time without penalty.
• Confidentiality and Anonymity: Confidentiality will be maintained by assigning pseudonyms to participants and companies. Identifying information will be removed from transcripts and reports. Data will be stored securely on password-protected devices; only the researcher can access raw data.
• Data Security: All electronic data will be encrypted, and physical documents will be stored in a locked cabinet. Data will be retained for a period consistent with ethical guidelines and institutional policies, after which it will be securely destroyed.
• Ethical Approval: Before commencing the study, ethical approval will be sought from the relevant institutional review board or ethics committee. The study will adhere to the Malaysian Insurance Institute's ethical principles and comply with industry-specific regulations.
• Professional Relationships: Given my professional background in the industry, care will be taken to avoid conflicts of interest. Participants will be assured that their participation is voluntary and will not impact professional relationships. Transparency about the researcher's role and the purpose of the study will be maintained throughout.

## Findings and analysis

### Overview of themes by research question

The analysis identified four main themes that collectively capture the core challenges influencing the adoption of AI-powered business intelligence solutions in the Malaysian insurance industry. These themes reflect organisational, technological, cultural, and regulatory complexities that participants confronted.

1. Regulatory Compliance and Data Security Concerns: Ensuring adherence to Bank Negara Malaysia’s guidelines, PDPA, and robust cybersecurity measures.
2. Talent Shortages and Skill Gaps: Addressing the lack of skilled AI professionals and the need for ongoing upskilling and training.
3. Cultural Resistance and Change Management Issues: Overcoming traditional work practices, employee scepticism, and the fear of job displacement.
4. Data Quality and Legacy System Integration Challenges: Managing inconsistent data sources, integrating AI-powered business intelligence solutions with outdated infrastructure, and implementing data governance frameworks.

**Regulatory compliance and data security concerns**

A central challenge highlighted by participants is ensuring compliance with local regulations, notably those set by Bank Negara Malaysia (BNM) and the Personal Data Protection Act (PDPA). Compliance is not perceived as a mere administrative requirement; instead, it functions as a strategic imperative that influences technology implementation choices and partnership decisions.

Participants frequently mentioned the RMiT framework issued by BNM, which mandates robust cybersecurity and risk management measures. While ensuring data integrity and customer trust, these regulations also impose stringent standards that can slow down the adoption of AI-powered business intelligence solutions.

Participant A underscored this necessity:

“Compliance is a cornerstone of our operations. We've established a dedicated risk management team that collaborates closely with our IT and AI departments to ensure all technological implementations meet regulatory standards, including the RMiT framework and PDPA.”

This sentiment was echoed by others who saw compliance as integral to maintaining market credibility and reducing long-term risks associated with data breaches and reputational damage.

### Talent shortages and skill gaps

The scarcity of skilled AI and data science professionals emerged as a major hurdle. Implementing AI-powered business intelligence solutions solutions requires specialized expertise to develop predictive models, manage complex data environments, and interpret advanced analytics. However, the local talent pool is often insufficient to meet these demands, compelling organisations to invest heavily in training, mentorship, and external collaborations.

Participant B described the difficulty in sourcing appropriate skills:

“Another significant challenge is the shortage of skilled professionals in AI and machine learning. We've addressed this by investing in internal capability development… but it's a continuous effort.”

This challenge affects implementation timelines and can limit the depth of AI applications within the organisation. Without the right human capital, even the most sophisticated AI tools may remain underutilised, inhibiting the full realization of potential benefits.

### Cultural resistance and change management issues

Cultural resistance to new technologies poses another substantive challenge. Long-standing work practices, scepticism toward AI’s reliability, and fears regarding job displacement can impede the integration of AI-powered business intelligence solutions into organisational workflows. Participants noted the importance of change management strategies emphasising clear communication, employee involvement, and tangible benefits to reduce apprehension and build trust.

Although cultural dimensions are often less tangible than technical or regulatory factors, they profoundly influence adoption outcomes. Participant D, reflecting on the human aspect of adoption, mentioned:

“We've used workshops, training sessions, and open communication channels to reduce resistance and build trust. Involving employees early and seeking their feedback helps a lot.”

By acknowledging cultural sensitivities and addressing them head-on, organisations can foster a more receptive environment where employees feel supported, valued, and ready to embrace AI-powered business intelligence solutions.

### Data quality and legacy system integration challenges

A recurring theme was the complexity of integrating AI-powered business intelligence solutions with legacy infrastructure, often compounded by inconsistent and fragmented data sources. Older systems not designed for advanced analytics can create isolated systems for data storage (commonly referred to as 'data silos'), hamper interoperability, and introduce data quality issues that undermine the accuracy of AI models.

Participant C highlighted these issues:

“Data quality is a significant hurdle due to inconsistencies and inaccuracies from various sources. To combat this, we've established rigorous data validation rules and automated cleansing processes.”

Addressing these technical challenges requires a concerted effort, including master data management strategies, standardized data formats, and middleware solutions to bridge old and new systems. By improving data reliability and ensuring seamless integration, organisations can enable AI tools to deliver meaningful insights, thereby reinforcing the business case for AI-powered business intelligence solutions.

### Summary of key findings

The study identified four main challenges shaping adopting AI-powered business intelligence solutions. First, ensuring regulatory compliance and data security emerged as a foundational concern. Insurers must align their technological implementations with frameworks like BNM’s RMiT and PDPA, influencing vendor selection, system configurations, and risk management procedures.

Second, talent shortages and skill gaps underscore a human capital constraint as the local market needs to supply sufficient AI and data science expertise. This gap constrains the pace and depth of AI integration, necessitating significant investments in training, partnerships, and internal capability-building.

Third, cultural resistance and change management issues reflect the human dimension of technological transitions. Traditional work practices, scepticism toward AI’s reliability, and fear of job displacement necessitate careful communication, employee involvement, and leadership commitment to foster a culture more receptive to digital innovation.

Finally, data quality and legacy system integration challenges highlight the technical complexity of aligning new AI-powered business intelligence solutions tools with older infrastructures and inconsistent data sources. Overcoming this barrier requires rigorous data governance, standardisation efforts, and thoughtful integration strategies.

### Limitations of the study

Based on interviews with nine participants, this qualitative case study provides in-depth insights but may limit generalizability. While the data offer rich contextual understanding, the experiences may only represent part of the Malaysian insurance industry or other regions. Additionally, the rapidly evolving nature of AI technology means that the relevance of these findings may shift as new solutions and regulatory frameworks emerge.

Further, as interviews rely on participant self-reporting, social desirability bias is possible. Participants may present their organisations in a favourable light or underemphasise challenges.

### Recommendations for future research

Future research could adopt a mixed-methods approach to combine qualitative depth with quantitative breadth, providing a more representative picture of the industry’s progress and challenges. Comparative studies across financial services sectors or international contexts could uncover unique cultural or regulatory dynamics. Additionally, longitudinal research would reveal how the adoption of AI-powered business intelligence solutions evolves, how skill gaps close over time, and how regulatory frameworks adapt to technological change.

## Conclusion

This study has examined the challenges associated with adopting AI-powered business intelligence solutions in the Malaysian insurance sector, interpreting the findings through the TOE and RBV frameworks. The results underscore that successful adoption is not solely about selecting advanced technologies—it also depends on aligning with external regulations, nurturing internal capabilities, ensuring data reliability, and creating environments where employees readily leverage AI insights.

By understanding and addressing these complex, interrelated factors, insurers can navigate the AI-powered business intelligence solutions landscape more effectively, enhancing operational efficiencies, strategic decision-making, and competitive positioning. This research contributes to a growing body of knowledge on AI adoption in emerging markets, offering practical and theoretical guidance for stakeholders seeking to harness the transformative potential of AI-powered business intelligence solutions.

### Ethical approval and consent

This study received ethical approval from the **University of Hertfordshire Ethics Committee on October 9, 2024** (Protocol No: **BUS/PGT/CP/06204**) and was conducted in compliance with institutional guidelines and adhered to the ethical principles outlined in the **Declaration of Helsinki** ( WMA, 2013). All necessary permissions for data collection were obtained prior to the study, and their informed consent was secured in accordance with ethical standards. Participants were fully informed about the purpose of the research, and their consent was secured in accordance with ethical standards. Confidentiality and data protection measures were strictly followed, and no invasive procedures were involved. Written informed consent was obtained before participation, ensuring that participants were fully aware of their rights, including the right to withdraw at any time without penalty

## Data Availability

The authors confirm that the data supporting the findings of this study are available within the article and extended data. Any additional data or supporting documents can be provided upon reasonable request. Access to the full dataset is **restricted due to ethical considerations** and **participant confidentiality**, as per the approval granted by the **University of Hertfordshire Ethics Committee (Protocol No: BUS/PGT/CP/06204, approved on October 9, 2024).** The Institutional Review Board has specified that data containing sensitive or identifiable participant information cannot be publicly shared. Researchers who wish to access the data may submit a formal request to the corresponding author, outlining the intended use of the data. Access will be granted only for academic and research purposes and under strict confidentiality agreements to ensure compliance with ethical guidelines. For further inquiries or data access requests, please contact ( sharmila.devi@newinti.edu.my). Figshare: EXTENDED DATA.doc. Doi: https://doi.org/10.6084/m9.figshare.28673729.v4 ( Ramachandaran S. (2025)) This project contains the following extended data:
• EXTENDED DATA.doc Data are available under the terms of the Creative Commons Zero “No rights reserved” data waiver (CC0 1.0 Public domain dedication).
