# Supplementary material for: Exploring the challenges of AI-driven business intelligence systems in the Malaysian insurance industry
Source: F1000Res. 2025 Apr 22;14:452. [Version 1] doi: 10.12688/f1000research.163354.1 (PMC12198725; doi:10.12688/f1000research.163354.1)
Supplement: Peer review history

# Reviewer report: Reviewer response for version 1

**DOI:** 10.5256/f1000research.179687.r392087

**Published:** 2025-06-25

**Authors:** vasantha shanmugam

**Affiliations:** 1 Vels Institute of Science, Technology & Advanced Studies (VISTAS), Chennai, India

The paper  effectively measured  its intended objectives .The Problem Statement and significance of research is clearly described with supporting literature.The author given clarity about the methodology of conducting research in briefly. But the sample size should have been increased to enhance the validity of the findings. But paper lacks detailed  discussion  to strengthen the analysis and findings.

 The study is limited by the coverage of  geographical area.But it may have impact on the generalizability of the findings.

Is the work clearly and accurately presented and does it cite the current literature?

Yes

If applicable, is the statistical analysis and its interpretation appropriate?

Partly

Are all the source data underlying the results available to ensure full reproducibility?

Partly

Is the study design appropriate and is the work technically sound?

Yes

Are the conclusions drawn adequately supported by the results?

Yes

Are sufficient details of methods and analysis provided to allow replication by others?

Partly

Reviewer Expertise:

Behavioural finance and operations

I confirm that I have read this submission and believe that I have an appropriate level of expertise to confirm that it is of an acceptable scientific standard.

# Reviewer report: Reviewer response for version 1

**DOI:** 10.5256/f1000research.179687.r380577

**Published:** 2025-05-13

**Authors:** Urvesh chaudhery

**Affiliations:** 1 Gitarattan International Business School (GIBS), New Delhi, India

**Summary -** The article explores the link between Human Resource Development (HRD) and organizational effectiveness in Nepalese manufacturing industries. It highlights HRD as a strategic mechanism vital to enhancing employee performance, organizational learning, and long-term business success. The author aims to assess the relationship between HRD practices—training, career development, performance appraisal, etc.—and organizational performance.

 Using a quantitative research design, data were collected through structured questionnaires from HR managers and supervisors across selected manufacturing industries in Nepal. The study employed statistical analysis to determine the correlation between HRD practices and indicators of organizational effectiveness such as productivity, employee satisfaction, and profitability.

- **Feedback**: While the topic of HRD and organizational effectiveness is important and relevant, especially in the context of developing economies like Nepal, the article does not sufficiently demonstrate how its findings provide new insights or go beyond existing literature.
- **Recommendation**: Emphasize the unique contributions of the Nepalese context and explicitly differentiate your findings from prior studies, particularly those conducted in other countries or industries.

Is the work clearly and accurately presented and does it cite the current literature?

Yes

If applicable, is the statistical analysis and its interpretation appropriate?

Yes

Are all the source data underlying the results available to ensure full reproducibility?

Yes

Is the study design appropriate and is the work technically sound?

Yes

Are the conclusions drawn adequately supported by the results?

Yes

Are sufficient details of methods and analysis provided to allow replication by others?

Yes

Reviewer Expertise:

Primary Domain: Business and Management StudiesSubdomain/Specialization: Human Resource Management (HRM) and  Human Resource Development (HRD)

I confirm that I have read this submission and believe that I have an appropriate level of expertise to confirm that it is of an acceptable scientific standard.
